# Supplementary material for: High-fidelity long-read sequencing of an avian herpesvirus reveals extensive intrapopulation diversity in tandem repeat regions
Source: PLoS Pathog. 2025 Aug 25;21(8):e1013435. doi: 10.1371/journal.ppat.1013435 (PMC12425393; doi:10.1371/journal.ppat.1013435)
Supplement: S1 File — Using MAFFT, we aligned the consensus genomes of CVI988-HiFi, HPRS-B14, Md5-HiFi, and 675A generated using PacBio HiFi, along with 39 additional consensus genomes belonging to strains from North America, Asia and Europe (see Table B in S1 File for accessions). A maximum-likelihood tree with gaps excluded was generated using the K3Pu+F + G4 substitution model, with bootstrap values ³70% shown. The tree is rooted at the midpoint. Arrows indicate newly assembled PacBio HiFi viral consensus genomes. Fig B: Multiple-sequence alignment of 40 UL36-PRRs reveals three distinct repetitive patterns. Graphical representation of the multiple sequence alignment used for phylogenetic analysis of the MDV049/UL36 proline-rich region (UL36-PRR) for 40 MDV strains with published consensus genomes (see Table B in S1 File for accessions). Amino acid sequences were initially aligned using MAFFT and then manually curated to improve alignment of the repeating units. The color-coding of strain names relates to the patterns and the tree shown in Fig 3C. Strains exhibiting Pattern 1 have names labelled in blue, Pattern 2 in pink, and Pattern 3 in green. MDV strains sequenced using PacBio HiFi are indicated with an arrow. Fig C: Structural variants of the a-like sequence harbor partial duplications of the pac-1 and pac-2 motifs. A) Using PacBio HiFi sequencing, several structural variants of the a-like sequence were identified. Common variants (>20% frequency in at least one strain) included reads with the “standard” 1 copy of the entire a-like sequence, as well as reads with a partial duplication of the a-like sequence involving the pac-1 (dark green), mTMR (dark yellow) and Uc segments (gray) (“Duplicated pac-1”). Rare variants included reads with any of the following: 2 or 3 copies of the entire a-like sequence; a partial duplication involving the pac-2 (green), mTMR and Uc segments (“Duplicated pac-2”); or 2 copies the entire a-like sequence interspaced by a partial duplication involving t [file ppat.1013435.s001.pdf]

S1 File: Supporting Information Figures and Tables

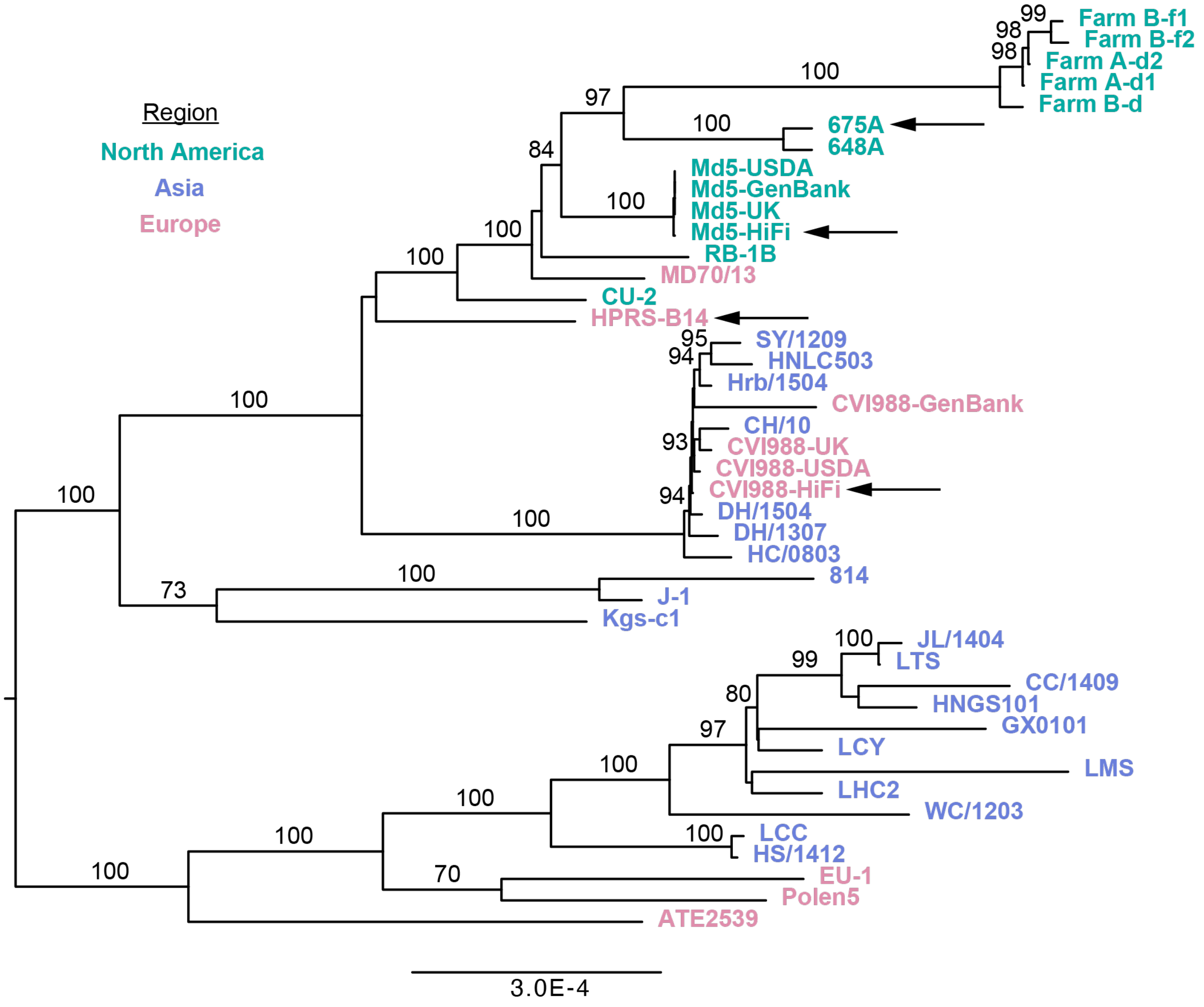

Fig A. PacBio HiFi consensus genomes cluster near previously published Illumina genomes of the same strain or near strains of the same pathotype. Using MAFFT, we aligned the consensus genomes of CVI988-HiFi, HPRS-B14, Md5-HiFi, and 675A generated using PacBio HiFi, along with 39 additional consensus genomes belonging to strains from North America, Asia and Europe (see **Table B** for accessions). A maximum-likelihood tree with gaps excluded was generated using the K3Pu+F+G4 substitution model, with bootstrap values ≥70% shown. The tree is rooted at the midpoint. Arrows indicate newly assembled PacBio HiFi viral consensus genomes.

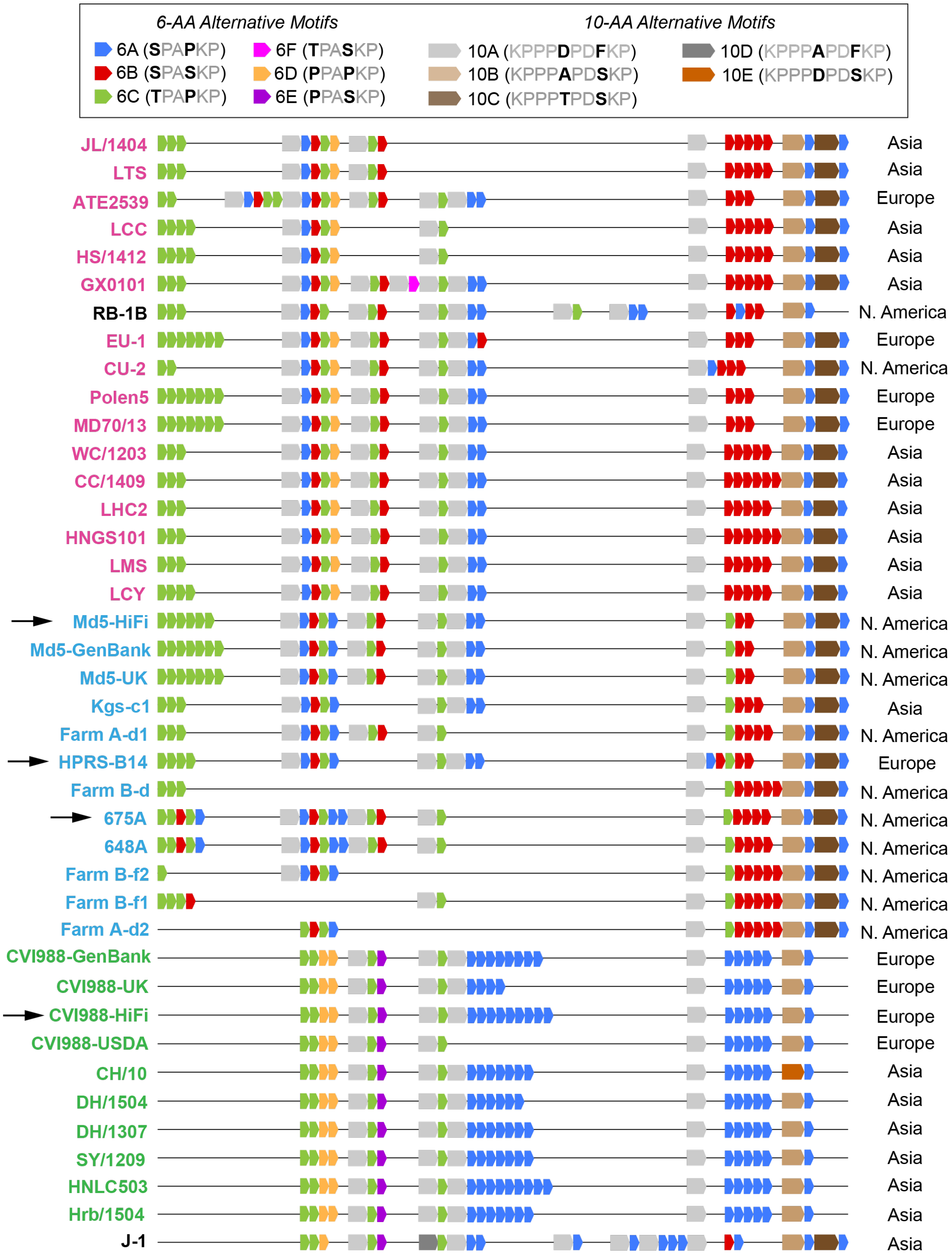

Fig B. Multiple-sequence alignment of 40 UL36-PRRs reveals three distinct repetitive patterns. Graphical representation of the multiple sequence alignment used for phylogenetic analysis of the MDV049/UL36 proline-rich region (UL36-PRR) for 40 MDV strains with published consensus genomes (see Table B for accessions). Amino acid sequences were initially aligned using MAFFT and then manually curated to improve alignment of the repeating units. The color-coding of strain names relates to the patterns and the tree shown in Figure 3C. Strains exhibiting Pattern 1 have names labelled in blue, Pattern 2 in pink, and Pattern 3 in green. MDV strains sequenced using PacBio HiFi are indicated with an arrow.

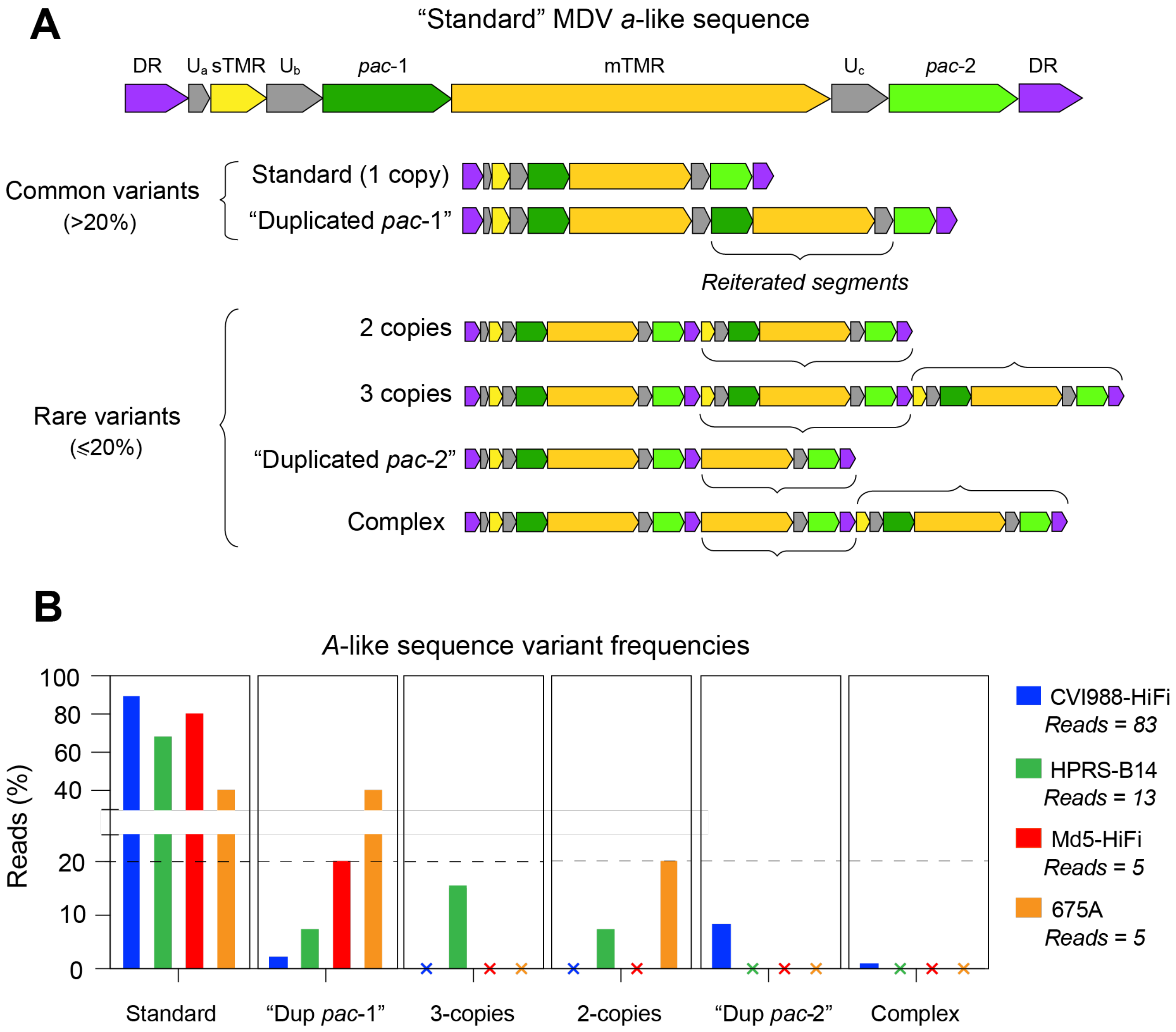

Fig C. Structural variants of the* a*-like sequence harbor partial duplications of the* pac*-1 and* pac*-2 motifs. A) Using PacBio HiFi sequencing, several structural variants of the *a*-like sequence were identified. Common variants (>20% frequency in at least one strain) included reads with the "standard" 1 copy of the entire *a*-like sequence, as well as reads with a partial duplication of the *a*-like sequence involving the *pac*-1 (dark green), mTMR (dark yellow) and U_c segments (gray) ("Duplicated pac-1"). Rare variants included reads with any of the following: 2 or 3 copies of the entire *a*-like sequence; a partial duplication involving the *pac*-2 (green), mTMR and U_c segments ("Duplicated pac-2"); or 2 copies the entire *a*-like sequence interspaced by a partial duplication involving the *pac*-2, mTMR and U_c segments ("Complex"). For each variant, duplicated segments are indicated with brackets below and/or above the sequence diagram. B) Bar graph showing the relative frequency of *a*-like sequence structural variants in the PacBio HiFi reads for each of the four strains. Variants present at frequencies >20% (dotted line) for at least one strain were classified as "common" variants, while variants below this threshold across all four strains were classified as "rare".

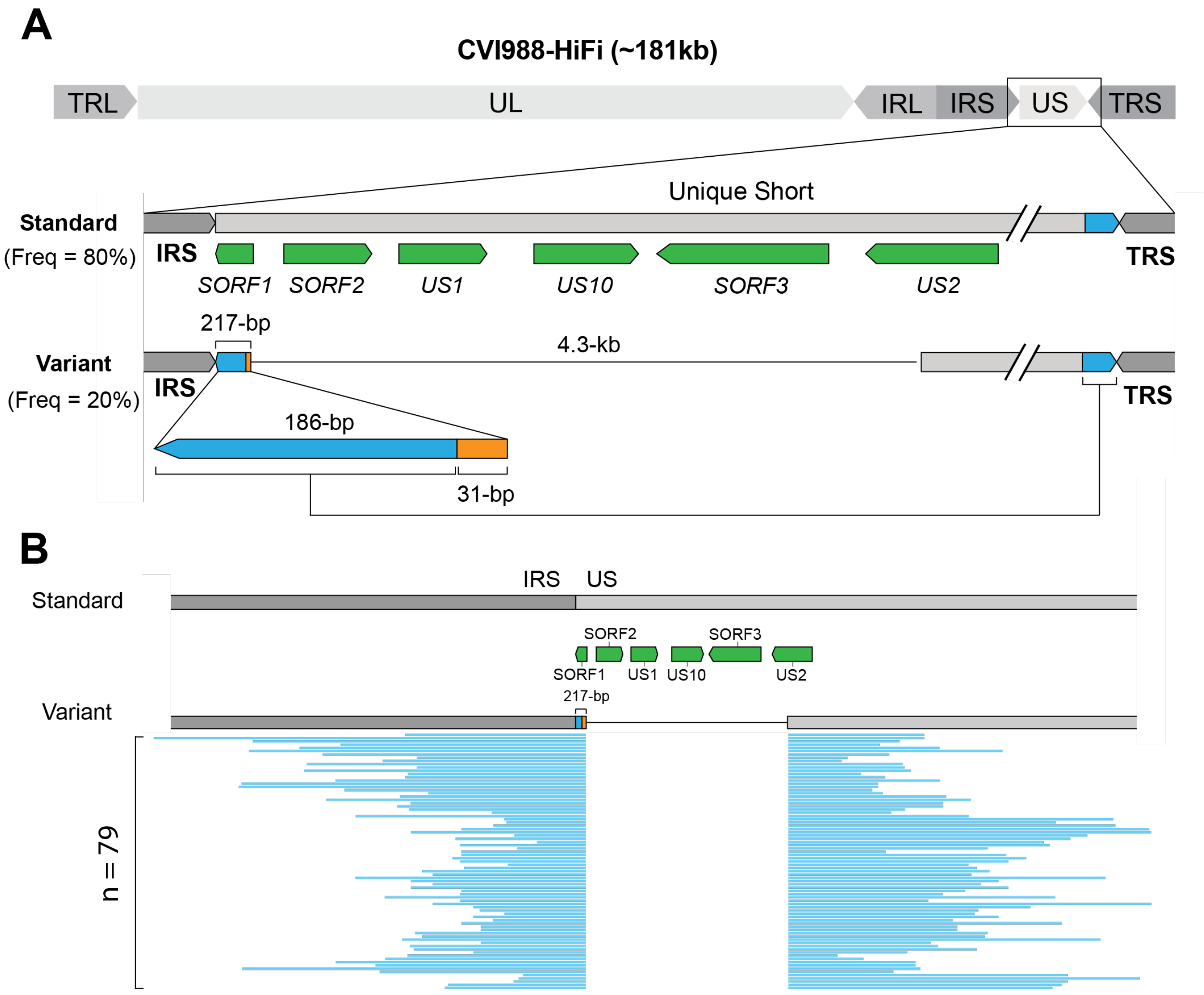

Fig D: PacBio HiFi reads enable detection of CVI988-HiFi variants with a 4.3-kb deletion in the Unique Short region. A) Graphical depiction of CVI988-HiFi variant showing an atypical US region with a large deletion and rearrangement. The first 186 nucleotides of this atypical US region correspond to the reverse complement of the last 186 nucleotides of a typical US region. The next 31 nucleotides do not map anywhere in the MDV genome. Thereafter, the reads have a 4.3 kb deletion compared to the majority of CVI988-HiFi reads, which removes SORF1, SORF2, US1, US10, SORF3, and approximately half of US2. B) The 79 PacBio HiFi reads supporting this US-deletion variant in the CVI988-HiFi sample exhibited a wide range of start- and end-points, suggesting that these variants may be part of otherwise intact genomes.

Table A: Number of PacBio HiFi reads used to assess intrapopulation diversity at each repetitive locus for all four strains

Region	CVI988-HiFi	HPRS-B14	Md5-HiFi	675A
MDV006.5	355	40	10	7
MDV075.2	329	38	11	26
UL36-PRR	640	43	8	26
mTMR (IRL/IRS)	83	13	5	5
LAT promoter	201	13	5	5
Meq-PRR (MDV076)	723	15	2	36

Table B. Published genomes and accessions for additional 39 included strains

**Strain**	**GenBank Accession**	**Publication Date**	**Length**	**PMID**	**Region**
Kgs-c1	LC589272	15-Oct-20	174,999	33228722	Asia
J-1	KU744555	18-Feb-17	176,118	27112385	Asia
814	JF742597	23-Feb-12	172,541	21984218	Asia
MD70/13	MF431495	12-Dec-17	177,844	29151863	Europe
648a	JQ806361	4-Apr-13	176,080	22923089	North America
RB-1B	EF523390	29-Oct-07	178,246	17721813	North America
CU-2	EU499381	5-Aug-08	176,922	18351449	North America
HNGS101	MG432697	6-Mar-18	175,888	29407378	Asia
JL/1404	KU744559	18-Feb-17	176,083	27112385	Asia
LTS	KU744557	18-Feb-17	176,023	27112385	Asia
CC/1409	KU744560	18-Feb-17	175,561	27112385	Asia
GX0101	JX844666	3-Dec-12	178,101	23166235	Asia
LHC2	MW247189	19-Apr-22	177,327	35266322	Asia
WC/1203	KU744558	18-Feb-17	176,057	27112385	Asia
LCY	KX290013	12-Apr-17	175,319	28194622	Asia
LCC	KU744556	18-Feb-17	175,525	27112385	Asia
HS/1412	KU744561	18-Feb-17	175,532	27112385	Asia
Polen5	MF431496	12-Dec-17	177,821	29151863	Europe
EU-1	MF431494	12-Dec-17	177,828	29151863	Europe
ATE2539	MF431493	12-Dec-17	177,868	29151863	Europe
LMS	JQ314003	1-Aug-12	177,526	22476905	Asia
HNLC503	MG518371	29-Apr-19	178,195	31698116	Asia
HC/0803	MW531728	10-Jul-22	174,808	35266322	Asia
SY/1209	MW247208	19-Apr-22	177,240	35266322	Asia
Hrb/1504	MW247204	19-Apr-22	177,839	35266322	Asia
DH/1307	MW247201	19-Apr-22	177,235	35266322	Asia
DH/1504	MW247202	19-Apr-22	177,991	35266322	Asia
CH/10	MW247200	19-Apr-22	177,401	35266322	Asia

CVI988	DQ530348	22-Mar-07	178,311	17374751	North America
Md5	NC_002229	13-Aug-18	177,874	10933706	North America
MDV-Md5-UK	PP032834	19-Feb-24	178,276	39036034	Europe
MDV-CVI988-UK	PP032835	19-Feb-24	176,532	39036034	Europe
MDV_Md5_vv_1977_MD	PP032832	19-Feb-24	176,724	39036034	North America
MDV_709B_v_2010_PA	PP032833	19-Feb-24	176,946	39036034	North America
Farm A-dust 2	KU173115	04-Jul-16	178,049	27747299	North America
Farm A-dust 1	KU173116	04-Jul-16	177,967	27747299	North America
Farm B-feather 1	KU173117	04-Jul-16	178,327	27747299	North America
Farm B-feather 2	KU173118	04-Jul-16	178,540	27747299	North America
Farm B-dust	KU173119	04-Jul-16	178,169	27747299	North America

Table C: List of previously reported and newly identified MDV 5'LAT-deleted molecular subtypes

Molecular Subtype	Deletion length (bp)	Present in CVI988$_{HiFi}$	PMID
A	32	Yes	25298182
-	82	Yes	-
-	118	Yes	-
A1	124	Yes	25298182
-	129	Yes	-
-	139	Yes	-
A2	145	Yes	25298182
-	182	Yes	-
-	203	Yes	-
B	233	No	25298182
B1	239	Yes	25298182
C	268	No	25298182
D	369	No	25298182
E	431	No	25298182
F	514	No	25298182
Gi	592	No	25298182
G	609	No	25298182
G2i	619	No	25298182
G3i	626	No	25298182
G4i	636	No	25298182
β	664	Yes	25298182
H	721	No	25298182
I	798	No	25298182
Ii	803	No	25298182
α	805	Yes	25298182
δ	838	No	25298182
N	859	No	25298182

ε	876	No	25298182
J	896	No	25298182
Ki	1047	No	25298182
K	1128	No	25298182
Li	1174	No	25298182
L	1292	No	25298182
M	1372	No	25298182
γ	1400	No	25298182

Table D. Dunn's test of mTMR island copy numbers, which relates to Fig 5B-E.

Dunn's multiple comparisons test	Min rank diff	Adjusted P Value	Z
CVI988 vs. HPRS-B14	50.41	<0.0001	5.504
CVI988 vs. Md5	16.70	>0.9999	1.181
CVI988 vs. 675A	59.90	0.0001	4.236
HPRS-B14 vs. Md5	-33.72	0.2216	2.087
HPRS-B14 vs. 675A	9.485	>0.9999	0.5870
Md5 vs. 675A	43.20	0.1567	2.224

Table E. Representative strains and accessions of the VL-Meq, S-Meq and VS-Meq isoforms

Strain	GenBank Accession	Publication Date	Isoform	Length	PMID
Italy/Ck/507/15	QBM00229	19-Mar-19	VL-Meq	1,257	30850833
Iraq3A	KC243262	03-Jul-13	S-Meq	897	23901776
Ck/IR/99-35/2021	MW990216	01-Oct-22	VS-Meq	798	34423692
