# Supplementary material for: High-fidelity long-read sequencing of an avian herpesvirus reveals extensive intrapopulation diversity in tandem repeat regions
Source: PLoS Pathog. 2025 Aug 25;21(8):e1013435. doi: 10.1371/journal.ppat.1013435 (PMC12425393; doi:10.1371/journal.ppat.1013435)
Supplement: Attachment — Submitted filename: Responses_to_Review_v2.pdf [file ppat.1013435.s002.pdf]

We appreciate the suggestions of the Reviewers and have incorporated these into the revised text. The *Reviewers' comments are included in blue italics* and are addressed point-by-point below (text in black). Each Reviewer's comments are presented in full, and organized by Reviewer for clarity. References recommended by Reviewer 1 have been shortened to first-author and DOI for brevity. **Page and line numbers below refer to Marked-up Manuscript file**, which highlights all changes from the original submitted version, including both the main text and the supplement. We appreciate the contributions of the review process and look forward to hearing back from you.

In line with a field-wide shift toward telomere-to-telomere assemblies, our main goal was to establish a foundational set of end-to-end or "complete" genomes for four isolates of Marek's disease virus (MDV), a herpesvirus that serves as a model of vaccine-driven virulence evolution [1–3]. The four viral strains we studied span the four known classes of virulence (i.e., "pathotypes") for this pathogen [2,3]. The unique contribution of this study is the observation that MDV is highly variable in tandem repeat regions, both within and between samples, despite the conventional wisdom that MDV harbors little genetic diversity [2,4]. This diversity was obscured in previous studies due to their reliance on short-read data, and poses new avenues for exploring the evolution of MDV virulence. While we did not seek to establish direct links between this variation and virulence in this initial study, future studies from our lab and others will certainly seek to test the hypotheses generated by this work. Our genomic characterization of cultured viral DNA provides a ground-truth readout of the viral stocks that are used to initiate *in vivo* empirical studies. These data will be critical to validate future studies that seek to develop enrichment-based amplification methods for long-read sequencing of direct-from-host viral samples [4,5]. We have now provided this critical context in the Abstract, Introduction, and Discussion sections (Lines 46-52, 111-114, 135, and 608-621 in the Marked-up Manuscript).

Reviewer #1, section 1: *This is a well written manuscript using long read sequencing to investigate heterogeneity in various pathotypes of marek's disease virus.*

We appreciate this evaluation.

Reviewer #1, section 2: *The authors confirmed the differences in many regions of the MDV genome that has been investigated over the last score using short read sequencing and PCR to determine the length of the amplicons encoding MDV repeat elements. The novel discover was a deletion in the CVI988 strain that represented 20% of the reads. The significance of this is unknowns since this could be the result of problems with tissue culture propagation. Furthermore, this region has been shown to be nonessential for oncogenicity. So, it's role in virulence is questionable. Here is the reference Parcells et al, [doi:10.1128/jvi.69.12.7888-7898.1995].*

The manuscript would be more impactful if the source of the MDV DNA was from lymphocytes purified from infected birds. This DNA could be captures using Modern hybiridation capture techniques. For reference Steiert et al [doi:10.1093/nargab/lqac051]

We appreciate the Reviewer's suggestion that hybridization capture techniques could provide a solution to this issue, however this type of enrichment for long-read sequencing has yet to be applied to viral pathogens in the milieu of abundant host DNA. Moreover, the protocol cited above used 24 PCR cycles to obtain sufficient sequencing depth, which increases the risk of bias, particularly in the context of repeat-region diversity [5]. Despite the relatively low coverage that we obtained for non-attenuated strains, our study implemented a sequencing protocol that did not rely on amplification, which minimized the risk of bias and provides a critical "ground truth" for future studies that aim to develop enrichment-based methods for long-read sequencing. We now include these points in the Discussion section (Lines 632-636 and 827-840 in the Marked-up Manuscript).

In addition to the four cultured stocks of MDV sequenced here, we also attempted to sequence four samples derived from peripheral blood lymphocytes (PBL) of infected birds, as the Reviewer suggested. However, due to the naturally occurring low concentrations of MDV DNA in samples from infected birds, only 0.002-0.003% of the resulting PacBio reads were viral. We have amended the Discussion to highlight the need to develop methods to better capture or enrich MDV DNA from field samples (Lines 827-837). As noted above, the present study provides an important baseline of un-amplified MDV genome and repeat-region characterization using long-reads, which future studies can build on to develop accurate and bias-free enrichment methods to capture viral genomes from *in vivo* samples.

The manuscript would be greatly enhanced if the author performed empirical experiments to validate the significance of their findings. For example, Silva & Gimeno [doi:10.1007/s11262-006-0022-7], Silva et al [doi:10.1128/jvi.78.2.733-740.2004]. This work indicated that expansion of the 132 occurs during serial passage induced attenuation, but other changes also occur that are involved in attenuation.

Another example of empirical data demonstrating the significance of Meq SNP in virulence is provides by Conradie et al. [doi:10.1371/journal.ppat.1009104]. A further example of the loss of function is provided by Kim et al. [doi:10.3390/v15112263]. This reviewer believes this is an excellent example of using the current long read sequencing technologies whether PacBIO or Minion to reinvestigate the heterogeneity of the large DNA virus MDV; but empirical data is needed to make this manuscript more impactful.

Please see our opening statement on the goals of this manuscript. We anticipate that future studies from our lab and others will seek to test the hypotheses generated by this work empirically. Prior studies that have empirically tested a given genomic region's impact on virulence for MDV – or any other herpesvirus – have done so in the context of a manuscript fully devoted to that topic. The necessary experiments entail the generation, authentication, and testing of multiple recombinant and rescue viruses in an appropriate *in vivo* model (e.g., [6]), which would exceed the scope of this manuscript – which already includes 9 figures and 6 tables.

With regard to the 132-bp repeats, we agree that preceding studies have suggested that these repeats alone cannot account for attenuation. However the above citations and related studies used MDV strains that did not contain the alternative version of the 132-bp repeat with a thymine in position 67 (132B) [7–10]. As such, the phenotypic impact of the 132B repeat remains largely unexplored. We have revised the Discussion to now highlight this point and related references (Lines 640-651).

Reviewer #1, section 3: *Sate the names of the US genes that were deleted in the CVI988 strains that represents 20% of the reads.*

We appreciate the Reviewer bringing this to our attention. While the names of the six genes affected by the 4.3-kb deletion in US (SORF2, US1, US10, SORF3, US2) were provided previously in the Results section, we have now included them throughout the manuscript (Lines 46, 128, 587-588, 797-798, and 1506-1513).

Reviewer #2, section 1*: This study uses long-read sequencing technology to look at the tandem repeat regions of MDV. Previous short read methods were not sufficient to sequence the entire regions of several isolates. They included multiple strains ranging in virulence from avirulent (vaccine strain) to highly virulent. The analysis suggests that genes in the repeat regions play in important role in virulence. The last statement in the abstract, that herpes virus repeats can have high diversity is not unique because this has been noted before with several other family members.*

The reviewer is correct that herpesviruses are well known to vary in terms of their repeat-length, however most prior reports have focused on assessing repeat diversity <u>between</u> strains (e.g., [11]), rather than examining repeat-diversity <u>within</u> a given strain (e.g., [12]). We have modified the abstract to clarify this point (Lines 46-52 in the Marked-up Manuscript).

This is very interesting work looking at genetic mechanisms in virulence and would also be relevant for generation of improved vaccine strains. More detailed followup studies should be interesting. Overall the manuscript is well written and the conclusions are supported by the data.

We appreciate this evaluation.

The major weakness is in the description of the methods and data presentation in a couple of places.

Specifically:

1. Line 204. They state "genetic distance comparisons" but it looks like they actually mean the substitution model that was used. This is the same issue for the next section.

2. Line 209. They need to justify the use of the specific model listed.

3. The bootstrap values in figure 3C are very low and the tree is not rooted-it needs to be rooted. This needs justification or please carry out the analysis. IqTree might be a better program to use.

4. Line 220. MLTree might be better and they don't justify the use of the Jukes-Cantor model over other models.

We appreciate these suggestions from Reviewer #2 and agree that our original phylogenetic methods warranted improvement. We have now replaced the phylogenetic analyses with maximum likelihood (ML) trees, using IQ-TREE and rooted trees as recommended. This entailed replacing Figure 3 (on UL36 repeat region) and supplementary Figure S1 (on whole genomes), and revising their figure legends (Lines 967-975 and 1446-1449). We provide explicit justifications for the substitution models used for each analysis in the Methods (Lines 241-244 and 253-255). The term "genetic distance comparisons" has been replaced in all instances by "phylogenetic analyses" to improve clarity (Lines 228, 246, 374, 415, and 552-558).

5. Page 15, lines 330-345. What are the criteria for separating the output into the clusters? Was a quantitative approach used or some other method?

We agree that the link between the phylogenetic analyses and the three "clusters" of UL36-PRR repeat types was not clearly defined. We now clarify that we identified three distinct "patterns" in the UL36-PRR, each characterized by specific repetitive sequence elements, which were identified by visual inspection of the aligned sequences (Lines 377-389). We further clarify that the ML tree serves to illustrate phylogenetic groupings and to address "atypical" strains such as RB-1B and J-1 which do not fit into these patterns (Lines 414-417).

6. Same section, would a linear regression analysis of virulence vs. copy number strengthen the argument?

Our findings for the UL36-PRR suggest that repeat composition (i.e., alternative motifs), as opposed to repeat length (i.e., copy number), drives the diversity of this locus. As such, a linear regression analysis based on copy number would not be suitable in this situation.

7. The discussion is lengthy and contains too much rehashing of the results. This should be tightened up.

We appreciate the Reviewer's recommendation for the Discussion section. We have now shortened this section and ensured that each paragraph only covers the most relevant information (e.g., text deleted at Lines 601, 640-650, 661-695, 701-705, 713, 787, 804-825, and 827-840).

Reviewer #2, section 2: *The methods need improvement as noted above. No new experiments are needed but the changes in the methods are not trivial.*

All of Reviewer 2's requested changes have been addressed in reply to their points above.

Reviewer #2, section 3*: None.*

Reviewer #3, section 1: *In this manuscript…, Renner DW. et al. used High-Fidelity Long-Read Sequencing to investigate genomic sequence differences within Marek's disease viruses (MDV) of different phenotypic strains: CVI988- HiFi (attenuated), HPRS-B14 (virulent), Md5-HiFi (very virulent), and 675A (very virulent). The total DNA of MDV-infected cells was used for DNA library preparation and sequenced by PacBio HiFi sequencing. They found 1) Out of the 4 investigated strains, the heavily passaged and attenuated vaccine strain CVI988-HiFi exhibits extensive intrapopulation diversity in the 132-bp tandem repeats overlapping the MDV006.5/MDV075.2 transcripts; 2) The proline-rich region of MDV049/UL36 (UL36-PRR) has six alternative versions of 6-AA repeating units, and is highly variable across MDV strains; 3) CVI988-HiFi variants completely lack the LAT promoter region and have deletions of variable sizes in the 5' end of LAT; 4) The length and composition of the multiple telomeric repeats (mTMR) of the MDV a-like sequence are highly polymorphic and unstable; 5) The proline-rich region (PRR) of the Meq oncoprotein has three distinct amino-acid repeating units; 6) 20% of the reads showed CVI988-HiFi variants had a 4.3-kb deletion in the Unique Short region.*

Overall, the High-Fidelity Long-Read Sequencing was able to reveal several genomic diversities in different MDV strains. These findings may provide a link between MDV tandem repeats and phenotypic traits like virulence and attenuation.

- *Although they found variations and polymorphic tandem repeats among different phenotypic strains, a direct link to strain phenotype will need experimental proof.*

We agree that establishing a direct link between the observed genotypes and viral phenotypes will require experimental proof. At the same time, we acknowledge that conducting the necessary assays would comprise a separate manuscript. As noted in above in reply to Reviewer 1, prior studies that have empirically tested the connection of a given genomic region to virulence for MDV have done so in the context of a manuscript fully devoted to the generation, authentication, and testing of recombinant and rescue viruses in an appropriate *in vivo* model (e.g., [6]). We have now revised the text to frame this work as a necessary first step to specifically tackle this question (Lines 49-50, 135, and 801-804 in the Marked-up Manuscript).

- *One major concern with this study is the viral genome sequence was obtained from the total tissue culture DNA, which may have a mixed infection in different stages.*

The replication of MDV in culture entails productive replication, and would not include additional stages such as latency or transformation. Both cultured and bird-derived MDV face the barrier of having limited viral DNA quantities in the milieu of host DNA, and potentially having different stages of viral replication co-mingled due to asynchronous infection. In addition *in vivo* field infections of MDV often entail a more complex mixture of vaccine and virulent viral strains (e.g., [13]), or MDV in the presence of additional avian pathogens (e.g., [14,15]). The cultured viral DNA used here provided sufficient DNA input for un-amplified PacBio long-read sequencing [5]. In addition, our characterization of cultured viral DNA here provides a ground-truth readout of same types of viral stocks that are used to initiate *in vivo* empirical studies. We have revised the text to highlight these points for the reader (Lines 135, 619-621, and 632-636).

- *The defective viral genomes (DVGs) or incomplete DNA replications may contribute to the variations and polymorphic tandem repeats seen in different phenotypic strains.*

We agree and have revised the Discussion to reflect these points (Lines 792-797 and 801-825).

- *Another concern is there were significantly more reads for the tissue culture-adapted strain CV1988-HiFi than for other strains.*

Non-attenuated strains of MDV like HPRS-B14, Md5, and 675A are notoriously difficult to grow to high titers in chicken embryo fibroblasts [3]. While previous studies have at times circumvented this issue by using duck embryo fibroblasts, this approach risks altering the genomic composition of the viral population in a way that is further removed from a live infection in commercial poultry [16]. Likewise, as noted in our reply to Reviewer 1, the use of enrichment-based approaches to overcome the limitations of low viral genome numbers for the more-virulent strains would introduce different concerns of PCR

bias, particularly in the context of intra-population length variation at the repeats. The limitations of the lower coverage for the more-virulent strains have been added to the Discussion (Lines 632-636).

It begs the question, do variations and polymorphic tandem repeats exist in the virion genomes?

Polymorphic tandem repeats have been previously reported *in vivo* using alternative approaches to whole-genome sequencing, particularly in the context of the 132-bp repeats of the MDV006.5/MDV075.2 transcripts. Silva *et al*. used Southern blotting to show that MDV DNA recovered from tumors in live birds contained a heterogenous population of viruses containing 3-16+ copies of the 132-bp repeats, while Volkening *et al.* used long-read RNA-Seq to identify MDV transcripts with 1-11 copies of the 132-bp repeats in chicken epithelial skin cells [17,18]. These prior studies have been incorporated into the Discussion (lines 647-651).

The following are specific issues to be addressed:

1. In terms of 132 A repeat, there are more reads for CVI988-HiFi than other stains; the Md5-HiFi has only two reads containing the repeat (Sup Table 1). Could this come from tissue culture variation.

As noted above, the lower number of PacBio reads for non-attenuated strains (i.e., HPRS-B14, Md5, and 675A) relate to the lower titers that can be achieved for their growth *in vitro* [3]. Since CVI988/Rispens was developed as a vaccine strain through multiple generations of passaging *in vitro*, its level of intrapopulation diversity may well be a result of this culture history [19]. This is reflected in the prior studies cited in Results and Discussion (Lines 70-72, 638-640, 714-751, and 801-804).

2. In Fig. 4E, ~14% of CVI988- HiFi reads still contained the 60-bp repeats, which means the portion of the viral genome still has the LAT promoter. Is there any evidence that CVI988- HiFi lacks LAT expression?

While we have not conducted experimental assays to measure LAT expression in CVI988-HiFi, prior studies have detected LAT expression in commercial batches of CVI988/Rispens vaccine [20,21]. We have modified the Discussion section to highlight these observations, and to propose that our discovery that a subset of CVI988-HiFi reads still retain a functional LAT promoter could help to explain these findings (Lines 748-751).

4. Lines 408-410: "For each strain, reads -----the majority 5'LAT molecular subtype (------) were manually realigned to optimize the mTMR alignment and visually inspected". What does the subtype imply here?

We apologize for this confusing phrasing, which referred to the need to optimize the local alignments at repeat regions, for PacBio reads that included both the a-like sequence and the nearby LAT promoter region. We have rephrased the text to clarify this point (Lines 489-499).

3. In the section on mTRM within the a-like sequence, Fig. 5 was not described correctly.

5. Line 414: Figure 5B should be Figures 5B-D.

6. Lines 416-421: There is no Figure 6 on mTMR

7. Lines 416-418: Again, there is no Figure 6 with information on mTMR at each stretch.

We appreciate the Reviewer for bringing these to our attention. We have now made these changes and ensured that all figures and tables are properly referenced in the manuscript (e.g., Lines 505, 508, 510).

8. A subset of 79 (20%) CVI988-HiFi reads from CVI988-HiFi had a 4.3-kb deletion in the Unique Short region. This means that the majority of the viral genome still has the 4.3-kb sequence. Could these reads come from defective viral genomes (DVGs) or incomplete or aborted replication?

Yes, these are potential explanations for the reads encompassing this deletion. We have revised the Discussion to explicitly mention DVGs as a potential explanation for the presence of the US-deleted CVI988-HiFi variants (Lines 801-825).

9. Line 315: "For CVI988-HiFi, a total of 640 reads were found to completely contain the 315 UL36-PRR (Supplementary Table 3)." Is this a different Sup Table 3? The Supplementary Table 3 submitted is on "Published genomes for additional 39 included strains."

We appreciate the Reviewer bringing this to our attention. This has been fixed (Line 359). The order of Supplementary Tables has also been corrected to reflect the order in which they appear in the manuscript, and all references to these tables have been updated accordingly.

***Reviewer #3, section 2**: 1. In Fig. 4E, ~14% of CVI988- HiFi reads still contained the 60-bp repeats, which means the portion of the viral genome still has the LAT promoter. Is there any evidence that CVI988- HiFi lacks LAT expression?*

As noted for this Reviewer's query #2 above (page 5), prior studies have detected LAT expression in commercial batches of CVI988/Rispens vaccine [20,21]. We have modified the Discussion section to highlight these observations, and to propose that our discovery that a subset of CVI988-HiFi reads still retain a functional LAT promoter could help to explain these findings (Lines 748-751).

2. Lines 416-421: There is no Figure 6 on mTMR,

We apologize for the mis-labeling. This has been corrected.

3. A subset of 79 (20%) CVI988-HiFi reads from CVI988-HiFi had a 4.3-kb deletion in the Unique Short region. This means that the majority of the viral genome still has the 4.3-kb sequence. Could these reads come from defective viral genomes (DVGs) or incomplete or aborted replication? Is there any evidence that US1, US10, and US3 are missing?

As noted in reply to query #8 above, we have modified the Discussion to include DVGs as a potential explanation for the presence of the US-deleted CVI988-HiFi variants (Lines 801-825). We also explicitly mention that the affected genes are ORF1, SORF2, US1, US10, SORF3, and US2 (US3 was not impacted), and have modified **Supplementary Figure 4** to reflect this (Lines 46, 128, 587-588, 797-798, and 1506-1513).

***Reviewer #3, section 3:** Make sure the figures and tables are properly referenced in the manuscript.*

We apologize the previous errors in select figure and table references. These have been fixed.

References cited above:

1. Read AF, Baigent SJ, Powers C, Kgosana LB, Blackwell L, Smith LP, et al. Imperfect Vaccination Can Enhance the Transmission of Highly Virulent Pathogens. Fraser C, editor. PLOS Biol. 2015;13: e1002198. doi:10.1371/journal.pbio.1002198
2. Dunn JR, Black Pyrkosz A, Steep A, Cheng HH. Identification of Marek's disease virus genes associated with virulence of US strains. J Gen Virol. 2019;100: 1132–1139. doi:10.1099/jgv.0.001288
3. Witter RL, Calnek BW, Buscaglia C, Gimeno IM, Schat KA. Classification of Marek's disease viruses according to pathotype: philosophy and methodology. Avian Pathol J WVPA. 2005;34: 75–90. doi:10.1080/03079450500059255
4. Ortigas-Vasquez A, Szpara M. Embracing Complexity: What Novel Sequencing Methods Are Teaching Us About Herpesvirus Genomic Diversity. Annu Rev Virol. 2024;11: 67–87. doi:10.1146/annurev-virology-100422-010336
5. Steiert TA, Fuß J, Juzenas S, Wittig M, Hoeppner MP, Vollstedt M, et al. High-throughput method for the hybridisation-based targeted enrichment of long genomic fragments for PacBio third-generation sequencing. NAR Genomics Bioinforma. 2022;4: lqac051. doi:10.1093/nargab/lqac051

6. Denesvre C, You Y, Rémy S, Vychodil T, Courvoisier K, Penzes Z, et al. Impact of viral telomeric repeat sequences on herpesvirus vector vaccine integration and persistence. PLOS Pathog. 2024;20: e1012261. doi:10.1371/journal.ppat.1012261
7. Niikura M, Dodgson JB, Cheng HH. Stability of Marek's disease virus 132-bp repeats during serial in vitro passages. Arch Virol. 2006;151: 1431–1438. doi:10.1007/s00705-006-0724-0
8. Becker Y, Tabor E, Asher Y, Davidson I, Malkinson M, Witter RL. PCR detection of amplified 132 bp repeats in Marek's disease virus type 1 (MDV-1) DNA can serve as an indicator for critical genomic rearrangement leading to the attenuation of virus virulence. Virus Genes. 1993;7: 277–287. doi:10.1007/BF01702588
9. Silva RF, Reddy SM, Lupiani B. Expansion of a Unique Region in the Marek's Disease Virus Genome Occurs Concomitantly with Attenuation but Is Not Sufficient To Cause Attenuation. J Virol. 2004;78: 733–740. doi:10.1128/JVI.78.2.733-740.2004
10. Silva RF, Gimeno I. Oncogenic Marek's disease viruses lacking the 132 base pair repeats can still be attenuated by serial in vitro cell culture passages. Virus Genes. 2007;34: 87–90. doi:10.1007/s11262-006-0022-7
11. Tyler SD, Peters GA, Grose C, Severini A, Gray MJ, Upton C, et al. Genomic cartography of varicella-zoster virus: a complete genome-based analysis of strain variability with implications for attenuation and phenotypic differences. Virology. 2007;359: 447–58. doi:10.1016/j.virol.2006.09.037
12. Jensen NJ, Depledge DP, Ng TFF, Leung J, Quinlivan M, Radford KW, et al. Analysis of the reiteration regions (R1 to R5) of varicella-zoster virus. Virology. 2020;546: 38–50. doi:10.1016/j.virol.2020.03.008
13. De Laney DB, Jones AE, Zerbes M, Tannock GA. Isolation of serotype 1 Marek's disease viruses from vaccinated Australian flocks. Vet Microbiol. 1995;46: 213–219. doi:10.1016/0378-1135(95)00085-O
14. Zhou J, Zhao G-L, Wang X-M, Du X-S, Su S, Li C-G, et al. Synergistic Viral Replication of Marek's Disease Virus and Avian Leukosis Virus Subgroup J is Responsible for the Enhanced Pathogenicity in the Superinfection of Chickens. Viruses. 2018;10: 271. doi:10.3390/v10050271
15. Li T, Xie J, Liang G, Ren D, Sun S, Lv L, et al. Co-infection of vvMDV with multiple subgroups of avian leukosis viruses in indigenous chicken flocks in China. BMC Vet Res. 2019;15: 288. doi:10.1186/s12917-019-2041-3
16. Nazerian K. Attenuation of Marek's disease virus and study of its properties in two different cell cultures. J Natl Cancer Inst. 1970;44: 1257–1267.
17. Silva RF, Reddy SM, Lupiani B. Expansion of a unique region in the Marek's disease virus genome occurs concomitantly with attenuation but is not sufficient to cause attenuation. J Virol. 2004;78: 733–740. doi:10.1128/jvi.78.2.733-740.2004
18. Volkening JD, Spatz SJ, Ponnuraj N, Akbar H, Arrington JV, Vega-Rodriguez W, et al. Viral proteogenomic and expression profiling during productive replication of a skin-tropic herpesvirus in the natural host. PLoS Pathog. 2023;19: e1011204. doi:10.1371/journal.ppat.1011204
19. Rispens BH, van Vloten H, Mastenbroek N, Maas HJ, Schat KA. Control of Marek's disease in the Netherlands. I. Isolation of an avirulent Marek's disease virus (strain CVI 988) and its use in laboratory vaccination trials. Avian Dis. 1972;16: 108–125.
20. Zhang Y, Tang N, Sadigh Y, Baigent S, Shen Z, Nair V, et al. Application of CRISPR/Cas9 Gene Editing System on MDV-1 Genome for the Study of Gene Function. Viruses. 2018;10: 279. doi:10.3390/v10060279
21. Jiang B, Wang J, Cao M, Jin H, Liu W, Cheng J, et al. Differential Replication and Cytokine Response between Vaccine and Very Virulent Marek's Disease Viruses in Spleens and Bursas during Latency and Reactivation. Viruses. 2022;15: 6. doi:10.3390/v15010006
