# Supplementary material for: High-fidelity long-read sequencing of an avian herpesvirus reveals extensive intrapopulation diversity in tandem repeat regions
Source: PLoS Pathog. 2025 Aug 25;21(8):e1013435. doi: 10.1371/journal.ppat.1013435 (PMC12425393; doi:10.1371/journal.ppat.1013435)
Supplement: Attachment — Submitted filename: Responses_to_Review_Second_Round_v2.pdf [file ppat.1013435.s003.pdf]

We appreciate the suggestions of the Reviewers and have incorporated these into the revised text. The *Reviewers' comments are included in blue italics* and are addressed point-by-point below (text in black). Each Reviewer's comments are presented in full, and organized by Reviewer for clarity. References recommended by Reviewer 3 have been shortened to first-author and DOI for brevity. **Page and line numbers below refer to Marked-up Manuscript file**, which highlights all changes from the original submitted version, including both the main text and the supplement. We appreciate the contributions of the review process and look forward to hearing back from you.

Reviewer #1: *The authors have responded appropriately to the issues raised by the reviewers.*

We appreciate Reviewer 1's initial feedback and appreciate their positive view of the revised manuscript.

Reviewer #2, section 1: *In this revised manuscript titled: High-Fidelity Long-Read Sequencing of an Avian Herpesvirus Reveals Extensive Intrapopulation Diversity in Tandem Repeat Regions, Renner DW. et al. addressed most of the concerns raised by the reviewers in the discussion section. The revision does not have any new data or significant improvement.*

Overall, this manuscript provided significant detail on the difference within the tandem repeat regions among different strains of MDV. However, it did not highlight the key features of tandem repeats that are unique to different strains. Fig. 1D is not informative about the heterogeneity of tandem repeats between attenuated strains and virulent strains. Fig. 1D could be used to summarize the differences in tandem repeats unique for different strains investigated here. Additionally, the discussion section could include a synthesis section on the various tandem repeats. Are there tandem repeats that could be used to predict the virulence of the virus? What kind of tandem repeats could be seen in attenuated or virulent MDV? What is the driving force for the heterogeneity in the tandem repeats between different strains or within a specific strain?

We appreciate and agree with Reviewer 2's suggestion that Figure 1D could be used to convey a summary of our findings about interstrain differences in tandem repeats. We have revised the summary in Figure 1D to convey which interstrain differences in tandem repeats were primarily driven by differences in length (i.e., repeat copy number), composition (i.e., presence or absence of specific motifs), or both. We also added an indication of whether each repeat was analyzed at the nucleotide or the amino acid level. Please see the revised image and legend for Figure 1D for these changes (lines 745-753, and 874). This figure now provides a useful guide to the deeper analyses of each repeat region, which are found in later figures.

In line with earlier feedback from Reviewers 1 and 3, we have refrained from over-extending Figure 1D to convey claims of an established pattern of repeats that differentiate attenuated or vaccine strains from virulent strains. However we have added a horizontal line to separate the vaccine strain data from the disease-causing strains, to better highlight these differences for the reader. As noted in response to the first round of reviews, our analyses are hypothesis-generating and lead to proposed experiments to test the potential connections between repeat-differences and virulence phenotypes. To address Reviewer 2's suggestion to propose these experiments in the Discussion, we have added a new paragraph summarizing these potential connections as future directions (lines 664-680, as well as 519-520 and 590-600).

Reviewer #2, section 2: *No new experiment is required.*

We appreciate this evaluation.

Reviewer #2, section 3: *Fig 1D should be revised to summarize the differences in tandem repeats unique for different strains investigated here.*

Reviewer 2's requested changes have been addressed in reply to their points above.

Reviewer #3, section 1: *The revised manuscript is improved, and the authors addressed my concerns. There are a few minor issues with the revised manuscript.*

Sentence 532-534, "In addition to observing a wide range of copy numbers (2-37 copies) in CVI988, we found that no length was supported by more than 12% of the reads mapping to either locus." I find this statement difficult to interpret. Is there a better way of stating this?

We appreciate Reviewer 3's feedback and we have amended lines 550-553 to be more clear:

> "More specifically, CVI988-HiFi reads fully containing the 132-bp repeats displayed a wide range of repeat copy numbers (2-37 copies), and the consensus (i.e., most frequent) copy number in each locus accounted for only 9-12% of the reads."

Sentence 568-570, "Notably, our new model suggests that both of the proline-rich regions found in the MDV genome (i.e., UL36-PRR, Meq- PRR) exhibit complex repetitive patterns that are highly variable across MDV strains." Please expand on this new model. Perhaps a figure is needed. Proline rich region variations in UL36 and RLORF 7 (meq) have been known for some time.

Our study is the first to describe the Meq-PRR as a complex tandem repeat region and the first to highlight the similarities between this locus and the UL36-PRR based on the complex architecture of their tandem repeat patterns. Prior studies, whose work we cited throughout the manuscript, were based on the original model proposed by Jones et al. which proposed "simple" tandem repeats [2]. We have modified and expanded the corresponding paragraph clarify these points. We now also include the citation proposed by the Reviewer regarding past observed variations in Meq [1]. Rather than adding an additional figure here, we incorporated into this paragraph the summary of the Meq-PRR found in Fig. 6A (and its global comparison in Fig. 6F), and the UL36-PRR in Figure 3A (and its global comparison in Fig. S2). In both cases, these descriptions of common motifs found across many strains is more encompassing of the global diversity of MDV strains than prior models. We have now amended this paragraph to reflect this and to direct the reader in this part of the Discussion to the prior works on each PRR (lines 593-600).

For meq, (please add the following reference):
Shamblin et al. [doi: 10.1016/j.vetmic.2004.06.007]

For UL36 (already added)
Spatz & Silva [doi: 10.1007/s00705-007-0992-3].

These references are both included in the revised manuscript as part of this Discussion (Lines 593-600).

Besides these minor issues, the data is well presented in a well written manuscript with beautiful figures.

We appreciate Reviewer 3's positive evaluation in this regard.

Reviewer #3, section 2*: None.*

Reviewer #3, section 3: *Minor issues with their meq and UL36 new model.*

All of Reviewer 3's requested changes have been addressed in reply to their points above.

References cited above:

1. Shamblin CE, Greene N, Arumugaswami V, Dienglewicz RL, Parcells MS. Comparative analysis of Marek's disease virus (MDV) glycoprotein-, lytic antigen pp38- and transformation antigen Meq-encoding genes: association of meq mutations with MDVs of high virulence. Vet Microbiol. 2004;102: 147–167. doi:10.1016/j.vetmic.2004.06.007
2. Jones D, Lee L, Liu JL, Kung HJ, Tillotson JK. Marek disease virus encodes a basic-leucine zipper gene resembling the fos/jun oncogenes that is highly expressed in lymphoblastoid tumors. Proc Natl Acad Sci U S A. 1992;89: 4042–4046. doi:10.1073/pnas.89.9.4042
